# Racial Disparities in Cognitive Functioning: The Mediating Role of Social Resources

**DOI:** 10.1093/geroni/igaa057.1099

**Published:** 2020-12-16

**Authors:** Kazi Sabrina Haq, Margaret Penning

**Affiliations:** University of Victoria, Victoria, British Columbia, Canada

## Abstract

Despite frequent recognition of disparities in cognitive functioning between White and non-White older adults, the pathways or mechanisms through which race affects cognitive functioning have yet to be elucidated. The research questions addressed in this paper are: 1) Is there a relationship between racial minority status and cognitive functioning in middle and later life? 2) To what extent do social resources (i.e., social support, social networks, and social participation) mediate the relationship between racial minority status and cognitive functioning? 3) Finally, drawing on intersectionality theory, if social resources do mediate the relationship between racial minority status and cognitive functioning, to what extent is this mediation effect moderated by the interaction of gender and Socioeconomic Status (SES)? Using cross-sectional data drawn from the Canadian Longitudinal Study on Aging (CLSA) with a sample of over 50,000 Canadians (2010-15) aged 45 to 85 years, multivariate regression analyses (OLS, logistic, multinomial logistic) assess the mediating effect of social resources on the relationship between racial minority status and cognitive functioning. Controlling for age, gender and other relevant determinants, preliminary results reveal that racial disparities in cognitive functioning (i.e., lower cognitive test scores) exist in Canada and that this relationship is partially mediated by some indicators of social resources (e.g., functional social support, emotional social support). Our findings suggest the need for interventions targeted at increasing social resources for racial minority groups to cope with the risk of developing cognitive impairment in later life.

